# Genomic Regions Associated With Salinity Stress Tolerance in Tropical Maize (*Zea Mays L.*)

**DOI:** 10.3389/fpls.2022.869270

**Published:** 2022-05-31

**Authors:** Pervez H. Zaidi, Mohammed Shahid, Kaliyamoorthy Seetharam, Madhumal Thayil Vinayan

**Affiliations:** ^1^Asia Regional Maize Program, International Maize & Wheat Improvement Center (CIMMYT), Hyderabad, India; ^2^International Centre for Biosaline Agriculture (ICBA), Dubai, United Arab Emirates

**Keywords:** maize, abiotic stress, salinity, SNP, association analysis

## Abstract

Being a widely cultivated crop globally under diverse climatic conditions and soil types, maize is often exposed to an array of biotic and abiotic stresses. Soil salinity is one of the challenges for maize cultivation in many parts of lowland tropics that significantly affects crop growth and reduces economic yields. Breeding strategies integrated with molecular approach might accelerate the process of identifying and developing salinity-tolerant maize cultivars. In this study, an association mapping panel consisting of 305 diverse maize inbred lines was phenotyped in a managed salinity stress phenotyping facility at International Center for Biosaline Agriculture (ICBA), Dubai, United Arab Emirates (UAE). Wide genotypic variability was observed in the panel under salinity stress for key phenotypic traits viz., grain yield, days to anthesis, anthesis-silking interval, plant height, cob length, cob girth, and kernel number. The panel was genotyped following the genome-based sequencing approach to generate 955,690 SNPs. Total SNPs were filtered to 213,043 at a call rate of 0.85 and minor allele frequency of 0.05 for association analysis. A total of 259 highly significant (*P* ≤ 1 × 10^–5^) marker-trait associations (MTAs) were identified for seven phenotypic traits. The phenotypic variance for MTAs ranged between 5.2 and 9%. A total of 64 associations were found in 19 unique putative gene expression regions. Among them, 12 associations were found in gene models with stress-related biological functions.

## Introduction

Increasing maize productivity is inevitable for plant breeders in the backdrop of meeting the ever-increasing demand for feeding the growing population and decreasing arable lands (Foley et al., 2011; Hansen et al., 2019). In addition to this socioeconomical stress, the wide adaptability of maize exposes it to several major abiotic stresses *viz.*, moisture availability such as drought and excessive moisture, and heat and salinity stresses (Zaidi et al., 2010; Lobell et al., 2011; Smale et al., 2011; Cairns et al., 2012; Enders et al., 2019). In recent years, salinity stress has been gaining momentum causing economically consequential yield losses in tropics (Bänziger and Araus, 2007). Unpredictable environmental variables resulting in increased rate of evapotranspiration due to rise in temperature, and increased inundation of land mass with sea water due to rise in their levels are some of the major factors responsible for changes in soil properties and making it more saline. In addition, unchecked exploitation of ground water is also among one of the key factors in increasing the incidence of salinity (Flowers and Yeo, 1995; Munns, 2002; Shahid et al., 2018). Recent estimates stated that globally, around 1,128 million ha of land was affected by salinity stress (Mandal et al., 2018), which is 20% of the cultivable area and 33% of the irrigated area (Shrivastava and Kumar, 2015).

The maize crop is reported to be moderately susceptible to salinity stress (Zörb et al., 2004; Chinnusamy et al., 2005). In general, all crop stages and all parts of maize are affected by salinity stress. In the early phase of crop development, saline affects seed germination by hampering water absorption by germinating seed due to Na^+^ and/or Cl^–^ toxicity that alters the osmotic potential of a soil solution (Hasegawa et al., 2000; Khajeh-Hosseini et al., 2003; Munns and James, 2003; Farsiani and Ghobadi, 2009). The early vegetative phase of the crop expresses stunted growth (Munns, 1993) with suppressed leaf expansion and internodal growth (Qu et al., 2012) due to reduction in cell elongation (Szalai and Janda, 2009) and programmed cell death. The reproductive phase of the crop is affected by micro-sporogenesis elongation of stamen filament, rate of photosynthesis, source-sink limitation, abortion of assimilation by fertilized kernels, which results in reduced kernel number and seed set, and, ultimately, reduced economic yield (Abdullah et al., 2001; Rahnama et al., 2010; Kaya et al., 2013).

Mitigating salinity stress through several agronomical and other management practices are available, but adopting those techniques by small-holderand marginal farmers will add up to their cost of cultivation and increase the risk of low returns and even crop failures in some cases, which challenges the sustainability of farmers to adopt those practices (Minhas and Sharma, 2003). Developing salt tolerance cultivars is considered relatively better, suitable for adopt to range of farmers with varied land-holdings and most economical approach. Maize genome being known to express its diversity for diverse environmental scenario (Hoisington et al., 1999) and its rich molecular marker, particularly SNPs resource is an added advantage to develop salt tolerant varieties or hybrids within short span of time through molecular breeding approach (Gilliham et al., 2017). Salt stress in *per se* and its tolerance mechanism in crops at physiological level, cellular level and phenotypical level are more complex (Farooq et al., 2015). In the maize crop, a number of QTLs were reported for rate of seed germination, salt tolerance ranking, shoot fresh and dry weights, shoot K^+^/Na^+^ ratio, and Na^+^ and K^+^ concentrations in shoots (Cui et al., 2015; Luo et al., 2017, 2021; Zhang et al., 2018; Xie et al., 2019). However, studies on salt tolerance of maize and reporting of QTLs/genomic regions for salt tolerance are still very limited in field-grown maize crop for a full crop cycle. Among several molecular breeding approaches, genome-wide association studies (GWAS) have several advantages (Frova and Sari-Gorla, 1994; Shikha et al., 2021). They take into account the historical recombination found in a broad panel of diverse germplasm and population-wide linkage disequilibrium (LD) among single nucleotide polymorphisms (SNPs) and quantitative trait loci (QTLs) (Luo et al., 2021). Furthermore, genome-wide scanning can locate genomic regions associated with several stress tolerance mechanism and nullify the complexity to a reasonable extent than relying solely on the phenotypic approach (Prasanna et al., 2021). With this rationale, this study was aimed at (i) managing the stress phenotyping of an association mapping panel consisting of 305 diverse maize inbred lines under saline conditions (ii) identifying genomic regions associated with grain yield and key secondary traits in maize under salinity stress using the GWAS approach.

## Materials and Methods

### Phenotyping

The association mapping panel consisting of 305 maize inbred lines used in this study is an amalgamation of advanced generation breeding lines derived from CIMMYT’s tropical and subtropical pools and populations from Latin America, Africa, and Asian maize programs. These lines were selected out of over 1,000 lines evaluated in Asian tropics for their general adaptation under optimal growing conditions. Lines with reasonably good adaptation in Asian tropics were selected for constituting the panel named CIMMYT Asia Association Mapping (CAAM), avoiding sister lines or over-representation of lines derived from any specific pools or populations. The inbred lines involved in this panel were derived from various pools and populations, including several biparental pedigree populations. Further details about the pools and populations of CIMMYT’s tropical maize program can be found elsewhere (Edmeades and Heisey, 1997; Zaidi et al., 2016a).

The panel was phenotyped the during dry season of 2017 and 2018 in a managed salt stress phenotyping facility at International Center for Biosaline Agriculture (ICBA) (25.0947°N, 55.3899°E), Dubai, United Arab Emirates (UAE), which is located about 23 km from the Arabian Gulf (Persian Gulf). The soils at ICBA experimental fields are sandy in texture, including fine sand (sand 98%, silt 1%, and clay 1%), calcareous (50–60% CaCO_3_ equivalents), porous (45% porosity), and moderately alkaline (pH 8.22). Saturation percentage of the soil is 26% with very high drainage capacity, while electrical conductivity of its saturated extract (ECe) is 1.2 dS m ^–1^. According to American Soil Taxonomy (Soil Survey Staff, 2010), the soil is classified as Typic Torripsamments, carbonatic, and hyperthermic (Shahid et al., 2009). A drip irrigation system was used for the field experiments with drippers at a 0.25-m distance and row-to-row spacing of 0.5 m. About 13.3 mm of water per day was used for irrigation. The field trials followed a lattice design, and each entry was planted in a 2-m row length with a plant-to-plant distance of 0.25 m and a row-to-row distance of 0.5 m with two replications. For the first 2 weeks, the experiment materials were irrigated with fresh water to avoid seed germination issues. Afterward, the field was irrigated with saline water with an ECe of 8 dS m^–1^. In the reproductive stage, days to 50% anthesis (AD), days of 50% silking (SD), anthesis silking interval (ASI), and plant height (PH) were measured using the standard phenotyping protocol for maize (Zaidi et al., 2016b). At maturity, ears were harvested separately from each plot, and grain yield (GY) was recorded on a per-plot basis. Final grain yield was calculated after adjusting for kernel moisture content at 12.5% for each plot and converted into tons per hectare (t ha^––1^). In 2018, in addition to grain yield, cob length (CL), cob girth (CG), and kernel number (KN) per cob were also measured by capturing digital photographs of harvested cobs of each plot. The ears were properly arranged and placed on a contrast background with equal spacing between the ears to avoid any overlapping. The ears were imaged using a digital camera at a uniform height. An image analysis was performed using the ImageJ software,^1^ open-source software using a script (ear analyzer) developed at CIMMYT (Makanza et al., 2018).

### Statistical Analysis

The linear model lmer from package lme4 of R using REML was used to calculate BLUEs and BLUPs, and variance components were estimated using the META-R (Multi-Environment Trial Analysis in R) software (Alvarado et al., 2020) in which replications, blocks, and environments were treated as random factors and genotypes as fixed factor. A cross-year analysis was conducted following the model described below:


$$Y_{i\,j\,k\,l}=\mu +E\,n\,v_{i}+R\,e\,p_{j}\,\left(E\,n\,v_{i}\right)+B\,l\,o\,c\,k_{k}\,\left(E\,n\,v_{i}\,R\,e\,p_{i}\right)+G\,e\,n_{1}+E\,n\,v_{i}\times G\,e\,n_{1}+\in_{i\,j\,k\,1}$$

where Y_ijkl_ is the trait of interest, μ is mean effect, Env_i_ is the effect of i^th^ environment, Rep_i_ is the effect of i^th^ replicate, Block_j_ (Env_i_Rep_i_) is the effect of j^th^ incomplete block within i^th^ replicate and environment, Gen_l_ is the effect of l^th^ genotype, Env_i_ and Env_i_ × Gen_l_ are the effects of i^th^ environment and environment by genotype interaction, and ∈ is the residue. Broad-sense heritability (*h*^2^) was estimated as follows:


$$h^{2}=\sigma_{g}^{2}/\sigma_{g}^{2}+\sigma_{g\times e}^{2}/n\,E\,n\,v\,s+\sigma_{e}^{2}/\left(n\,E\,n\,v\,s\times n\,r\,e\,p\,s\right),$$

where $\sigma_{g}^{2}$, $\sigma_{g\times e}^{2}$, $\sigma_{e}^{2}$, nEnvs, and nreps represent variance estimates of genotype, genotype × environment interaction, error, number of environments, and number of replications, respectively. Descriptive statistics including mean, minimum, maximum, and Co-efficient of variation were also generated using standard procedures implemented in META-R. Best linear unbiased estimators (BLUEs) from individual year and across years were used for the genome-wide association studies (GWAS).

### Association Mapping Panel Genotyping

DNA extraction was conducted following the modified CTAB method (CIMMYT Applied Molecular Genetics Laboratory, 2003) on 3–4 week young leaves harvested from each inbred line involved in the panel. Single nucleotide polymorphisms (SNPs) were generated through GBS v2.7 using Illumina Hi-seq 2000/2500 at the Institute for Genomic Diversity, Cornell University, Ithaca, NY, United States. Physical coordinates of GBS SNPs were derived from AGPv2. A total of 955,690 SNPs were generated after imputing missing data points by accepting a 5% mismatch with the closest neighbor in small SNP windows across the entire maize database (∼22,000 *Zea* samples). The criteria for filtering SNPs for GWAS and LD (linkage disequilibrium) analysis were based on Suwarno et al. (2015) but with slight modifications. SNPs were filtered based on a call rate (CR) > 0.85 and with a minor allele frequency (MAF) ≥ 0.05 for the association analysis.

### Linkage Disequilibrium Analysis and Genome-Wide Association Studies

Linkage disequilibrium was estimated using 1, 28, and 913 SNPs filtered from the total SNPs with a CR of > 0.9 and a MAF of > 0.1. The extent of genome-wide linkage disequilibrium was estimated based on adjacent pairwise *r*^2^ values and the physical distance among the SNPs using the “nlin” function in R (Remington et al., 2001; R Development Core Team, 2015). Average pairwise distances in which LD decayed at *r*^2^ = 0.2 and *r*^2^ = 0.1 were then calculated based on the model given by Hill and Robertson (1968).

The SNPs (955,690) obtained for the association panel after imputation were further filtered to 213,043 SNPs using the multiple selection criteria of CR > 0.85 and MAF ≥ 0.05 for the GWAS analysis. The association between the filtered SNPs and the trait of interest was detected by employing a mixed linear model (MLM) in the SNP and Variation Suits v8.6.0 software (GoldenHelix, Inc., Bozeman, MT, United States^2^). The fitness of the model was determined by observing Q-Q (quantile-quantile) plots (Supplementary Figures 1, 2). A marker-trait association (MTA) with a *p-value* threshold of ≤ 10^–5^ was considered significant. Significant SNPs that fell within or near gene models (> 1 MB) were searched for the putative gene function in maize GDB in B73 RefGen_V3.^3^ Gene models having a stress related gene/biological function were considered as candidate genes.

## Results

The GWAS panel exhibited a highly (*P* > 0.001) significant genotypic variance for all the traits studied under salinity stress in both individual and across years. Environmental and genotype × environmental interaction variance was also significant for all the traits. The magnitude of genotypic variance and error variance was narrow for individual years, whereas the magnitude of error variance and G × E variance of across year analysis was multi-folded when compared to genotypic variance. Broad-sense heritability of the traits in 2017 and 2018 ranged from 0.4 (CL) to 0.9 (AD), whereas across-year heritability ranged between 0.2 (GYG) and 0.8 (AD) (Table 1). In addition, the standard deviation (SD) of traits during 2017 and 2018 fell within a desirable range for all the traits (Table 1).

**TABLE 1:** Descriptive statistics of the association mapping panel evaluated under salinity stress.

	Year	GY	AD	ASI	PH	CG^#^	CL^#^	KN^#^
Mean	2017	2.74	78.08	7.86	144.55			
	2018	3.54	70.51	6.43	173.11	4.57	11.41	3593.20
	Across	3.14	74.30	7.14	158.84			
Range	2017	1.38–3.71	66.27–90.72	1.18–16.15	124.66–163.83			
	2018	2.45–4.69	61.83–77.92	5.31–8.97	160.15–187.09	4.21–5.11	10.53–12.44	2770–4444
	Across	2.79–3.41	65.80–81.51	6.20–8.87	142.2–174.53			
SD	2017	0.17	1.08	0.93	8.00			
	2018	0.60	1.53	1.52	10.80	0.22	0.92	530.06
	Across	0.48	1.32	1.26	9.50			
Heritability	2017	0.67	0.98	0.91	0.73			
	2018	0.64	0.89	0.40	0.50	0.53	0.41	0.53
	Across	0.22	0.80	0.28	0.68			
Genotype Variance	2017	0.19	26.34	4.26	88.69			
	2018	0.25	9.72	0.76	57.99	0.03	0.30	156929.93
	Across	0.04	12.62	0.55	61.71			
G × E variance	Across	0.18	5.32	1.96	12.39	–	–	–
Error variance	2017	0.19	1.17	0.87	64.06			
	2018	0.29	2.37	2.34	116.67	0.05	0.86	280964
	Across	0.24	1.75	1.59	90.30			

*GY, grain yield; AD, days to 50% anthesis; ASI, anthesis silking interval; PH, plant height; CG, cob girth; CL, cob length; KN, kernel number; CV, coefficient of variance,.*

*^#^- traits recorded only in 2018.*

Mean GY, AD, and ASI in the panel was consistent between individual years and across years. The mean value of GY and ASI was < 4 tha^–1^ and > 5 days, respectively, with a very wide range for both traits (GY: 1.38–4.69 tha^–1^, ASI: 1.18–16.15 days). The mean of PH slightly varied between years and across years, but it had a comparatively narrow range. The low values of GY and higher values of ASI indicate the impact of salinity stress on the reproductive stage of the crop. A similar effect of salinity stress was found on other morphological traits as well.

**FIGURE 1:**
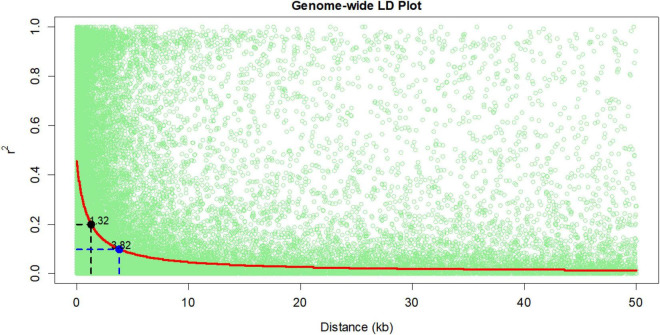
Linkage disequilibrium (LD) decay for the association mapping panel.

In this study, total SNPs (955 K) were shortlisted to 128,913 SNPs (CR = 0.9 and MAF = 0.1) for linkage disequilibrium (LD) decay estimation, and 213,043 SNPs (CR = 0.85 and MAF = 0.05) were used for the association analysis. The LD decay of the panel was 3.82 kb at *r*^2^ = 0.1 and 1.38 kb at *r*^2^ = 0.2 (Figure 1). In the association analysis for the seven phenotypic traits, a total of 259 highly significant (*P* < 1 × 10^–5^) (Supplementary Figures 3, 4) MTAs were identified for the traits under consideration. The phenotypic variance (PV) for MTAs ranged from 9.4 to 5.2%. For reproductive traits viz., GY, AD, and ASI, and 57 MTAs were found with PV ≥ 6%. A maximum number of 69 MTAs were identified for AD (total of 2017, 2018 and across) followed by ASI (59 MTAs) and grain yield (48 MTAs) (Table 2). In individual year, a maximum number of MTAs were identified for AD (28 MTAs in 2017) followed by GY, ASI, and CL (23 MTAs) (28 MTAs in 2018). In across-year GWAS, a maximum number of 25 MTAs were identified for AD. The traits GY, AD, and ASI had a maximum number of MTAs on chromosome 5 followed by chromosomes 2, 3, and 9 for KN, PH, and CL, respectively (Table 2 and Supplementary Table 1). A total of 15, 8, and 5 MTAs were found to be common between the across-year analysis and any of the individual year for AD, ASI, and PH, respectively (Table 2 and Supplementary Table 2). The SNPs, viz., S5_69435496, S5_69435513, and S5_69435514 were found to have common MTAs for GY and AD in 2017 and across year. Similarly, the SNPs S6_157029418 and S6_87402488 were found to be associated with GY and KN in 2018 (Table 3).

**TABLE 2:** Details of marker-trait associations identified for the traits under salinity stress.

	GY	AD	ASI	PH	CG	CL	KN
**2017**	23	28	23	12	–	–	–
**2018**	14	16	19	11	13	23	12
**Across**	11	25	17	12	–	–	–
**Total MTAs**	**48**	**69**	**59**	**35**	**13**	**23**	**12**
**P-values range**	2.4E-6- 9.0E-5	2.1E-6- 9.9E-5	7.1E-7- 9.9E-5	5.4E-6- 9.5E-5	2.9E-6- 8.7E-5	1.1E-7- 9.8E-5	2.0E-5- 9.8E-5
**PV range**	5.2–7.5	5.2–7.6	5.–8.2	5.2–7.2	5.2–7.4	5.2–9.4	5.2–6.1
**No. of Common SNP** (between across and any one year)	0	15	8	5	–	–	–
**Chromosome number** in which maximum SNP associated*	5 (11)	5 (20)	5 (15)	3 (8)	5 (5)	9 (5)	2 (3)

GY, grain yield; AD, days to 50% anthesis; ASI, anthesis silking interval; PH, plant height; CG, cob girth; CL, cob length; KN, kernel number. *Values in parentheses are the number of single nucleotide polymorphisms (SNPs) associated in the chromosome, PV, phenotypic variance.

**TABLE 3:** Details of marker-trait associations common for multiple traits.

S.No	Trait and year	Marker	Position in Mb	Chrom No	Gene name
1	GY-2017, AD-Across, AD-2017	S5_69435496	69.435	5	GRMZM2G117012_T01
2	GY-2017, AD-Across, AD-2017	S5_69435513	69.436	5	GRMZM2G117012_T01
3	GY-2017, AD-Across, AD-2017	S5_69435514	69.436	5	GRMZM2G117012_T01
4	GY-2018, KN-2018	S6_157029418	157.029	6	
5	GY-2018, KN-2018	S6_87402488	87.402	6	GRMZM2G114337_T01

In this study, a total of 236 MTAs were found in 113 unique putative gene expression regions. In particular, 11 MTAs: 7 for GY and 4 for AD were found in gene models with stress-related biological functions (Table 4). Notably the SNP S4_83197497, S6_87402488, S7_15827091, S5_191376709 in the gene model GRMZM2G168002, GRMZM2G114337, GRMZM2G478965, GRMZM2G057386 coding for the biological functions like glk49 - G2-like-transcription factor 49, Core-2,AP2-EREBP-transcription factors, which plays important roles in regulating various physiological functions or response to the various abiotic and biotics stress.

**TABLE 4:** List of marker-trait associations within the expression region of genome.

S.No	Trait and year	Marker	Chro No	Gene model name	Putative biological function
1	GY-Across	S4_83197497	4	GRMZM2G168002	glk49 – G2-like-transcription factor 49
2	GY-2018	S1_85990034	1	GRMZM2G453832	Mgt-6 magnesium transporter 6
3	GY-2018	S3_187169134	3	GRMZM2G036099	Cupin
4	GY-2018	S4_190375543	4	GRMZM2G013271	ofp22 – OVATE-transcription factor 22
5	GY-2018	S6_87402488	6	GRMZM2G114337	Core-2/
6	GY-2018	S7_15827091	7	GRMZM2G478965	ereb166 – AP2-EREBP-transcription factor 166
7	GY-2017	S5_191376709	5	GRMZM2G057386	ereb107 – AP2-EREBP-transcription factor 107
8	AD-Across	S1_250893506	1	GRMZM2G014392	vp14
9	AD-Across	S6_157569199	6	GRMZM2G305115	crt3 – Calreticulin3
10	AD-2017	S1_154507157	1	GRMZM2G092652	U-box domain-containing protein 13
11	AD-2017	S1_251143915	1	GRMZM5G859316	Expansins

## Discussion

Maize, a widely adopted crop under a range of climatic conditions, is often planted on salt-affected fields, as many farmlands are salinized (Zaidi et al., 2020; Prasanna et al., 2021). Analyzing the salt tolerance mechanism in maize by studying the diverse maize germplasm and understanding the genetic architecture of salinity tolerance can guide breeding programs aimed at developing salt-tolerant maize varieties (Luo et al., 2017). Apart from use of various agronomical and other biological means of avoiding/circumventing salinity stress, genetic intervention is considered a more viable and sustainable approach. Exploring the diversity and genotypic variability of the association mapping panel for salinity tolerance and identifying genomic regions associated with grain yield under salinity stress may significantly contribute to the development of salt-tolerant maize hybrids.

In this study, mean values of the traits were consistent between and across years, and a very wide range and magnitude of genotypic variance was observed between individual year and across years for each of the traits, making the panel very amenable for GWAS analysis. The LD decay in the panel based on 1, 28, 913 and SNPs was 3.82 and 1.34 kb at *r*^2^ = 0.1 and 0.2, respectively, which is characteristic of tropical and subtropical maize lines. The high LD (Yan et al., 2009) indicated that the panel is genotypically diverse in nature and favorable for obtaining high-resolution mapping (Yan et al., 2009; Suwarno et al., 2014; Yuan et al., 2019). A similar result of high LD decay was reported in previous association mapping panels developed for biotic and abiotic stresses and quality traits (Zaidi et al., 2016a; Hindu et al., 2018; Rashid et al., 2018; Seetharam et al., 2021).

The wide range of phenotypic expression for all the traits indicated that the genotypes responded in different intensities or adopted different physiological mechanisms and pathways to cope with salinity stress. Under salinity stress, the maize crop experiences high osmotic stress because of low water potential (Sümer et al., 2004), ion toxicity (Turan et al., 2010), and nutrient imbalance (Shahzad et al., 2012). In particular, each part of the plant is affected in different ways; for instance, leaf growth and expansion and internodal elongation are affected by accelerated abscission (Akram et al., 2010; Qu et al., 2012), and stomatal conductance is affected by altered water relationships and increased synthesis of abscisic acid, which in turn affects transpiration, photo-assimilation, and sink strength, reduces acid invertase activity in developing maize grains (Farooq et al., 2015), and ultimately reduces kernel set and seed weight (Abdullah et al., 2001; Kaya et al., 2013). Exploiting the existing genetic diversity by integrating the available advanced molecular approach can catalyze the process of developing salinity-tolerant inbred lines and hybrids.

In this GWAS study, 259 significant MTAs were found for seven phenotypic traits. The PV % explained by these traits was small (5.2–9.4%) and lower than the observed broad-sense heritability explained based on phenotypic data. This gap, also known as the missing heritability (Manolio et al., 2009; Eskin, 2015), might be the result of non-heritable genome changes, cross-talk between genes, RNAs, environments, statistical limitations, and many other unexplained factors (Slatkin, 2009; Marian, 2012; Zuk et al., 2012; Grandjean et al., 2013). Grain yield and related traits, viz., ASI and AD, had a total of > 50 MTAs (sum of individual and across-year analysis) but only a very few SNPs, i.e., 28, were significantly associated in individual and across years. Reduced commonality might be the result of the statistical threshold that is used in this study. Many associations that actually exist could have been missed reaching significance in any of the experiments. The frequency of “false negatives” depends on the number of genotypes and their diversity. The problem is very noticeable in sample sizes less than 500 compared to fewer samples (Beavis, 1997). This reduced commonality of SNPs indicates the complexity and influence of environment and its interaction with genome. A similar result of uncommon SNPs across location and year was reported by Seetharam et al. (2021) in maize for heat stress-related trait association analysis. Apart from this, notably, SNPs S5_69435496, S5_69435513, S5_69435514, S6_157029418, and S6_87402488 had across-year and multi-trait associations (GY, AD, and KN). The SNPs reported in this study were also found to be in QTL regions reported in earlier studies. Particularly the SNPs S5_163119125, S5_165071094, and S9_153775211, which were found to be significantly associated with AD-across and SNP S10_147245009, which was significantly associated with PH-across, were in the reported QTLs *qSPH5-1* (plant height at saline field), *qPHI9*, and *qPHI10* (plant height-based salt tolerance index) (Luo et al., 2017). Similarly, the SNPs S1_202305536 (GY-across), S4_235453550 (AD-across), and S7_168994991 (CL-2018) were found to be in QTLs reported for various seedling stage traits like shoot length, root length, root and shoot dry weight in a panel consisting of 348 maize inbred lines (Luo et al., 2021). This multi-trait associated SNPs and SNPs in previously reported QTL regions are of major importance, as they have a potential utility in routine marker-assisted selection/screening in breeding pipeline post-validation.

Associations (MTAs) in the expression region of genomes are of practical interest. In this study, 19 unique putative expression regions with various biological functions were associated with 64 SNPs. Plants develop a complex defensive or escape mechanism to overcome multiple stresses during the crop period through interconnected and diverse downstream signaling cascades by regulating genes (Papdi et al., 2015). A few SNPs that were in gene models (listed in Table 4) were found to a have biological function that is important for stress tolerance. The SNP S4_83197497 was in the gene model coding for glk49–G2-like-transcription factor 49. This G2-like (Golden 2 like) transcription factor plays a role in regulating chloroplast development to optimize photosynthetic capacity under varying environmental conditions (Waters and Langdale, 2009). Other SNPs, viz.- S7_15827091 and S5_191376709, were associated with coding regions for the AP2/EREBP transcription factor. Bodies of literature state that AP2/ERFs are strong regulators of several stress responsive genes and complex networks of pathways in response to stress stimulants in different crop growth stages (Licausi et al., 2013; Li et al., 2015). Similarly, the SNP S6_157569199 codes for crt3, calreticulin3. Calreticulin is a highly conserved and abundant multifunctional protein that is encoded by a small gene family and is often associated with biotic and abiotic stresses (Xiang et al., 2015). Calcium is an essential secondary messenger that mediates plant responses to developmental and environmental clues like salt stress. CRTs play an important role in regulating calcium signaling, assisting protein folding (Jia et al., 2009), etc. Furthermore, some of the SNPs were found associated with regions coding for cupin (S3_187169134), ofp22 (S4_190375543), vp14 (S1_250893506), U-box domain-containing protein-13 (S1_154507157), and expanins (S1_251143915). These regions have been reported to have major role in regulating several pathways in various growth and developmental stages of the crop in response to various stress inducers (Messing et al., 2010; Marowa et al., 2016; Hu et al., 2018). The SNPs/genomic regions identified in this study will be an effective resource to the maize breeding community to catalyze the process of maize varietal or hybrid development with stress tolerance. In addition, separate association analyses can be carried out for various growth stages viz., germination, emergence and early growth stages in same population to conclude with common genomic regions associated with the vegetative and reproductive stages of the crop. Significant SNPs in expressive regions can also be scanned for putative candidate regions associated with different crop stage salinity stress tolerance or pathways that can be utilized for candidate gene approach and followed up by deep sequencing of specific genes identified.

## Data Availability Statement

The datasets presented in this study can be found in online repositories. The names of the repository/repositories and accession number(s) can be found in the article/Supplementary Material.

## Author Contributions

PZ and MS: conceptualization, investigation, and supervision. KS and MV: formal analysis. PZ, MS, and KS: methodology. PZ, MS, KS, and MV: visualization and writing – review and editing. KS, PZ, and MV: writing – original draft. All authors contributed to the article and approved the submitted version.

## Conflict of Interest

The authors declare that the research was conducted in the absence of any commercial or financial relationships that could be construed as a potential conflict of interest.

## Publisher’s Note

All claims expressed in this article are solely those of the authors and do not necessarily represent those of their affiliated organizations, or those of the publisher, the editors and the reviewers. Any product that may be evaluated in this article, or claim that may be made by its manufacturer, is not guaranteed or endorsed by the publisher.

## Footnotes

1
https://imagej.nih.gov/ij/features.html

2
www.goldenhelix.com

3
https://www.maizegdb.org
